# Cytotoxic T cells modulate inflammation and endogenous opioid analgesia in chronic arthritis

**DOI:** 10.1186/s12974-017-0804-y

**Published:** 2017-02-06

**Authors:** Uta Baddack-Werncke, Melanie Busch-Dienstfertig, Sara González-Rodríguez, Santhosh Chandar Maddila, Jenny Grobe, Martin Lipp, Christoph Stein, Gerd Müller

**Affiliations:** 1 Department of Tumor Genetics and Immunogenetics, Max-Delbrück-Center of Molecular Medicine (MDC), Robert-Rössle-Strasse 10, 13125 Berlin, Germany; 2 Department of Anesthesiology and Critical Care Medicine, Charité Campus Benjamin Franklin, Hindenburgdamm 30, 12203 Berlin, Germany; 3 Current address: DLR project management agency, Department for Health Research, Heinrich-Konen-Str. 1, 53227 Bonn, Germany; 4 Current address: Instituto de Biología Molecular y Celular (IBMC), Av. de la Universidad s/n. Edif. Torregaitán, Elche, 03202 Alicante, Spain; 5 Current address: Santhosh Nursing Home, Darsi, Prakasam District, Andhra Pradesh 523247 India

**Keywords:** Rheumatoid arthritis, CD8 cells, Opioid peptides, Analgesia

## Abstract

**Background:**

This study examined the development of chronic pain, a cardinal symptom of rheumatoid arthritis (RA), in mice with antigen- and collagen-induced arthritis (ACIA). Since the role of CD8^+^ T cells in arthritis is controversial, we investigated the consequences of CD8-depletion on arthritis development and opioid modulation of pain in this novel model of chronic autoimmune arthritis.

**Methods:**

Disease severity in control and CD8-depleted animals was determined by histological assessment of knee-joint sections and measurement of autoantibody formation. Pain was evaluated by measuring mechanical allodynia and thermal hyperalgesia in von Frey and Hargreaves tests, respectively. The production and release of endogenous opioids and inflammatory cytokines was assessed in immunoassays.

**Results:**

In ACIA, mice display persistent mechanical allodynia and thermal hyperalgesia for more than 2 months after induction of arthritis. The blockade of peripheral opioid receptors with naloxone-methiodide (NLXM) transiently increased thermal hyperalgesia, indicating that endogenous opioid peptides were released in the arthritic joint to inhibit pain. CD8^+^ T cell depletion did not affect autoantibody formation or severity of joint inflammation, but serum levels of the pro-inflammatory cytokines TNFα and IL-17 were increased. The release of opioid peptides from explanted arthritic knee cells and the NLXM effect were significantly reduced in the absence of CD8^+^ T cells.

**Conclusions:**

We have successfully modeled the development of chronic pain, a hallmark of RA, in ACIA. Furthermore, we detected a yet unknown protective role of CD8^+^ T cells in chronic ACIA since pro-inflammatory cytokines rose and opioid peptide release decreased in the absence of these cells.

## Background

Chronic disorders with an inflammatory component are the most common diseases of aging and represent our greatest health threat [1]. Such disorders include rheumatoid arthritis (RA), a debilitating condition with significantly reduced quality of life characterized by severe pain, autoimmune responses, and joint inflammation [2]. Significant progress has been made regarding anti-inflammatory therapies, especially in the field of monoclonal antibodies against several proinflammatory cytokines such as IL-6, TNFα, and IL-1 [3–5].

However, chronic pain is still an unresolved clinical problem [6]. Standard analgesic treatments such as nonsteroidal anti-inflammatory drugs, opioids, or steroids exhibit detrimental side effects including sedation, respiratory depression, and an enhanced risk for gastrointestinal ulcers, bleeding, myocardial infarction, stroke, and infections [7, 8]. Thus, there is an urgent need for a better understanding of chronic pain conditions in adequate animal models to provide insights into cellular and molecular mechanisms of ongoing ache.

Antigen- and collagen-induced arthritis (ACIA) is a new mouse model that displays key features of RA such as chronic joint inflammation, cartilage and bone erosion, and prominent autoimmune responses against autologous collagen II, and anti-citrullinated peptide/protein antibodies (ACPA) that can be observed in the majority of RA patients [9]. ACPA are early markers for RA that can be detected years before disease onset [10, 11] and are associated with more severe joint destruction [12, 13]. With regard to immune cells, particularly CD8^+^ cytotoxic T cells (CTLs) have been shown to contribute to tissue destruction in autoimmune diseases. Given these similarities to the human disease, we set out to investigate if ACIA also resulted in a “chronic pain” phenotype and to identify cell types and molecules that play a role in pain sensing.

The immune system is linked to the nervous system by pro-inflammatory cytokines that directly or indirectly enhance the sensitivity of inflamed tissue towards painful stimuli (hyperalgesia) [14, 15]. In addition, immune cells produce anti-inflammatory cytokines and can potently reduce pain by the liberation of opioid peptides such as enkephalins, endorphins, and dynorphin [16–18]. Such mechanisms have been predominantly investigated in acute inflammatory injuries of animals and humans, but their relevance in chronic inflammation is not yet well understood. Murine colitis models of T cell-induced and DSS-induced colitis start shedding light on the regulation of pain by T lymphocytes [19, 20].

It is widely assumed that some T cell subpopulations play a pro-nociceptive role. However, several studies indicate that T cell deficiency increases the sensitivity towards painful stimuli in rodent models of acute visceral [21], inflammatory [22, 23], and neuropathic pain [24].

This raises the question if lymphocytes and, more specifically CD8^+^ CTLs, exert opioid-mediated antinociception under chronic inflammatory conditions. This led us to investigate the “pain” profile and opioid-mediated anti-nociception in ACIA in relation to histological parameters and to CTL depletion.

## Methods

### Mice

Female BALB/c mice were purchased from Charles River (Sulzfeld, Germany) and Janvier (France). Mice were used at 10–14-weeks of age. All animal studies were approved by the state ethics committee (Landesamt für Gesundheit und Soziales, Berlin, Germany) and performed according to institutional and state guidelines, under specific pathogen-free conditions.

### ACIA model

BALB/c mice were immunized by subcutaneous (s.c.) injection of 100 μg methylated bovine serum albumin (mBSA, Sigma-Aldrich, Schnelldorf, Germany) in 50 μl PBS emulsified with 50 μl complete Freund’s adjuvant (CFA, Sigma-Aldrich), followed 1 week later by a second s.c. immunization with mBSA plus bovine type II collagen in incomplete CFA [9]. One week later, mice were immunized s.c. with 50 μg mBSA and 100 μg bovine collagen type II (mdbioproducts, Zurich, Switzerland) in 50 μl PBS emulsified with 50 μl Freund’s incomplete adjuvant (IFA, Sigma-Aldrich). In parallel to each immunization step, 200 ng of *Bordetella pertussis* toxin (PTx, Calbiochem, La Jolla, CA, USA) were injected intraperitoneally (i.p.). Fourteen days later, on day 0, arthritis was induced by intraarticular (i.a.) injection of 50 μg mBSA dissolved in 20 μl PBS into one knee joint (ipsilateral) during brief isoflurane (Abbot, Wiesbaden-Delkenheim, Germany) anesthesia. The same volume of the vehicle (PBS) was injected into the contralateral knee. An “induction-only” control group of animals were not immunized with s.c. mBSA, but instead received s.c. PBS emulsified with adjuvant. On day 0, mBSA was injected into the ipsilateral and PBS into the contralateral knee joint.

**Table:** 

Treatment	ACIA	Induction-only control
s.c. immunization, day -21	mBSA + CFA	PBS + CFA
s.c. immunization, day -14	mBSA + IFA	PBS + IFA
i.a. ipsilateral knee, day 0	mBSA	mBSA
i.a. contralateral knee, day 0	PBS	PBS

### Depletion of cytotoxic T cells

To deplete cytotoxic T cells (CTL), ACIA mice received i.p. injections of 90 μg anti-CD8 [25] (Leinco Technologies, Inc., St. Louis, MO, USA), diluted in 200 μl sterile saline. The antibody was injected every 9–10 days starting 3 days before i.a. mBSA injection. Control animals received the isotype-matched control antibody (Leinco Technologies, Inc.). The amount of antibody was titrated so that depletion would last until the end of the study period (i.e., 60 days after arthritis induction).

### Immunohistochemical analysis

Animals were sacrificed and both knee joints were fixed in 4% buffered formaldehyde, decalcified with EDTA, and embedded in paraffin. Serial sections (4–5 μm thick) were cut and stained with hematoxylin and eosin (H&E) for microscopic evaluation. Scoring of the knee sections was performed in a blinded manner in three to four sections per knee joint. Two different scores were used, one for the severity of inflammation and one for the degree of destruction. Inflammation was scored by the degree of synovial hyperplasia (0–3 points), the amount of mononuclear cells infiltrating the synovial membrane (0–3 points), and fibrosis (0–3 points). Destruction of the joint was scored according to the level of cartilage and bone destruction (0–3 points) and pannus formation (0–3 points). Zero points in any of the categories corresponded to the absence of the parameter, 3 points indicated the strongest pathological phenotype, and points for each parameter were added up to the overall score. The experimenter was not aware of the preceding treatment of the animals.

### Assessment of nociceptive behavior

#### von Frey test

Mechanical hypersensitivity “allodynia” was assessed by applying von Frey filaments (Stoelting Co., Wood Dale, IL) as previously reported [26]. Briefly, animals were habituated to the test cages daily starting 6 days prior to behavioral testing. Mechanical allodynia was evaluated with calibrated von Frey filaments applied to the plantar surface of the hind paws using an up-down method [27]. The following calibrated von Frey filaments were used: 0.078 mN (0.0056 g), 0.196 mN (0.0076 g), 0.392 mN (0.041 g), 0.686 mN (0.059 g), 1.569 mN (0.14 g), 3.922 mN (0.28 g), 5.882 mN (0.54 g), 9.804 mN (0.66 g), 13.725 mN (1.15 g), 19.608 mN (2.35 g), and 39.216 mN (4.37 g) (Stoelting, Wood Dale, IL). The filaments were applied to the plantar surface of the hind paws for about 3 s until they bowed. The up-down method [28] was used to estimate 50% withdrawal thresholds. Testing began using a 3.922-mN (0.28 g) filament. If the animal withdrew the paw, the just-preceding weaker filament was applied. In the case of no withdrawal, the next stronger filament was applied. The maximal number of applications was six to nine, and the cut-off was 39.216 mN (4.37 g). Uninjured paws were typically withdrawn at the next filament (58.82 mN or 5.3 g), according to our previous studies [26].

#### Hargreaves test

The latency required to elicit paw withdrawal in response to radiant heat applied to the plantar surface of a hind paw from underneath a glass floor with a high intensity light bulb was measured with an electronic timer (IITC Inc. Life Science, Woodland Hills, CA). The stimulus intensity was adjusted to yield a baseline paw withdrawal latency of 10–12 s in healthy animals. The average of two measurements per paw assessed within a 1-min interval was calculated in seconds. To avoid tissue damage, a cut-off of 20 s was employed.

#### Behavioral protocols

Animals were habituated to the test cages daily starting 6 days prior to behavioral testing and were randomly assigned to the different treatment groups. Treatment assignments and preparation of syringes were performed by another person to assure experiment was performed in a blinded manner. Baseline mechanical and thermal thresholds were assessed once before each immunization step. The animals were tested daily during the acute phase (2–7 days after i.a. injections), followed by weekly testing until the end of the observation period (day 64).

Local endogenous opioid-mediated pain control was evaluated by injecting i.a. naloxone-methiodide (NLXM) (1 μg/10 μl) under isoflurane anesthesia. Control mice received solvent. The effect on heat sensitivity was assessed at 15 and 30 min after drug application using the Hargreaves test.

### Immunoglobulin production and ELISA

For the analysis of serum components, mice were bled retro-orbitally at regular intervals. Analysis of antigen-specific IgG titers or cytokine levels in the serum was performed with capture ELISA. To measure mBSA-specific IgG, immunostep microtiter plates (Nunc, Langenselbold, Germany) were coated with 2 μg per well mBSA in PBS and washed and incubated with diluted serum. Bound serum antibodies were detected using horseradish peroxidase-conjugated rabbit anti-mouse serum (Southern Biotech, Eeling, Germany). For the detection of ACPA, the anti-MCV (mutated citrullinatedvimentin) ELISA (OrgentecDiagnostica, Mainz, Germany) was used. Serum TNFα level was determined with the mouse TNFα DuoSet ELISA (R&D systems). To detect the IL-17A level in the serum, the purified TC11-18H10.1 antibody (BioLegend) served as capture antibody, in conjunction with the biotinylated TC11-8H4 (BioLegend) as detection antibody and recombinant murine interleukin 17 (IL-17) (BioLegend).

### Detection of opioid peptides

Ipsilateral knee joints from CTL-depleted and isotype-treated arthritic mice were dissected in the late chronic phase, i.e., >day 60. Knee joints were cut in half and subjected to enzyme digestion using RMPI-1640 medium supplemented with 3 mg/ml hyaluronidase and 1 mg/ml collagenase II (both Sigma-Aldrich) for 30 min at room temperature. The knee joint was grinded, and tissue homogenates were filtered twice using 70- and 40-μm cell strainers, respectively. Cell suspensions were washed in pure RMPI medium, spun down for 10 min at ×350*g*, and resuspended in 250 μl of pure medium. Cells were kept for 1.5 h at 37 °C under agitation; the supernatants were harvested after spinning and kept at −80 °C. The amounts of liberated opioid peptides were determined using a Methionine-enkephalin radioimmunoassay (RIA, Bachem, Peninsula Laboratories, USA) and a fluorescent human/rat beta-endorphin enzyme immunoassay (EIA, Phoenix Peptides) as previously described [29]. The EIA kit is validated by the manufacturer to be 100% cross reactive for murine endorphin. For RIA, 100 μl of supernatants were analyzed in duplicates. The remaining supernatants were analyzed in duplicate using EIA. Both assays are suitable to detect opioid peptides at the pg-level.

### Statistical analysis

All data were documented using Microsoft Excel 2003 for Windows (Microsoft Corporation). GraphPad Prism Version 5.01 for Windows (GraphPad Software, Inc.) software was used for statistical analyses. Data were analyzed for equal variance and for normal distribution using the D’Agostino & Pearson omnibus normality test or Kolmogorov-Smirnov test. Normally distributed independent data were analyzed by the Student *t* test and nonparametric independent data by the Mann-Whitney *U* test if only two groups were compared. To compare two groups of normally distributed dependent data, the paired *t* test was used. In case of nonparametric dependent data, the Wilcoxon test was applied. Multiple measurements of normally distributed data were analyzed by the one-way repeated measurements ANOVA or by the Kruskal-Wallis test in case of not normally distributed data. Post hoc comparisons were performed by Bonferroni’s multiple comparison test for normally distributed data and by Dunn’s method for not normally distributed data. Two-way repeated measurements ANOVA was used for two factor analyses followed by Bonferroni’s multiple comparison test. Statistical significance was usually considered if *P* < 0.05. For the analysis of repeated measurements, a Bonferroni correction was performed in MS Excel to calculate *t* values with a stepwise bisection of alpha starting with 0.05 according to the equation *t* = TINV (alpha; degrees of freedom). The residual degrees of freedom (DF), as calculated in the test by GraphPad Prism V5, was used for these calculations. Then, *t* values obtained from the Bonferroni multiple comparison tests were compared to the corrected *t* values. If *t*_(Bonferroni’s multiple comparison test)_ was larger or equal to the corrected *t* value, the differences were considered significant. Correlations of data were performed using the Spearman correlation.

## Results

### Evaluation of chronic arthritis

Once the mice had reached the chronic phase of arthritis, i.e., day 60 after induction, their joints were analyzed histologically by hematoxylin and eosin (H&E) staining.

**Fig. 1:**
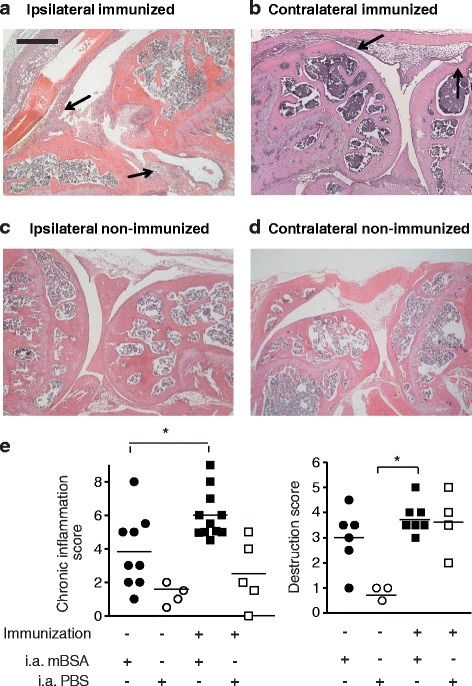
Histology of knee joint inflammation. H&E-stained knee joint sections of immmunized (**a**, **b**) and non-immunized (**c**, **d**) mice were analyzed 60–70 days after i.a. injection of mBSA (**a**, **c**) or PBS (**b**, **d**). *Arrows* indicate mononuclear cells infiltrating the synovial membrane and fibrosis, *black bar* 250 μm. **e** (*left panel*), the magnitude of joint inflammation was determined in a 3 parameter scoring system for synovial hyperplasia (1–3 points), infiltration of the synovial membrane by mononuclear cells (1–3 points), and fibrosis (1–3 points). **e** (*right panel*), the joint destruction score was determined by adding up pannus formation (1–3 points) and cartilage and bone erosion (1–3 points). Statistical analysis was performed using the Mann-Whitney *U* test to compare the scores between mBSA-injected knee joints of immunized (*N* = 11) and non-immunized (*N* = 9) animals (*P < 0.05); the Wilcoxon signed rank test was used to compare the scores between mBSA- (ipsilateral) and PBS-injected (contralateral) knee joints

In addition to the comparison of ipsilateral (i.a. mBSA induction) vs. contralateral (i.a. PBS) joints of the same animals, we also examined a control group (induction-only) that was not immunized s.c. but had only received i.a. mBSA (tissue sections Fig. 1a–d). As expected, the induction-only mice had significantly lower inflammation scores when compared with ACIA mice (Fig. 1e, left panel). The incidence of scores ≥4 was 100% in ACIA mice, but less than 50% in induction-only mice. Higher scores (≥6) were observed in 45% of the ACIA mice (*N* = 11) but only in 11% of induction-only animals (*N* = 9).

Next, we compared ipsilateral with contralateral (i.a. PBS) knee joints. The latter also developed inflammation in later stages of the disease. Ipsilateral joints showed higher severity scores than contralateral ones, but these differences were not statistically significant.

Scores for joint destruction were highest for the ipsilateral knees of ACIA mice (Fig. 1e, right panel). Scores ≥4 were observed in 42% of the ACIA mice (*N* = 7) but only in 14% of induction-only animals (*N* = 6).

### Correlation of pain with parameters of inflammation

**Fig. 2:**
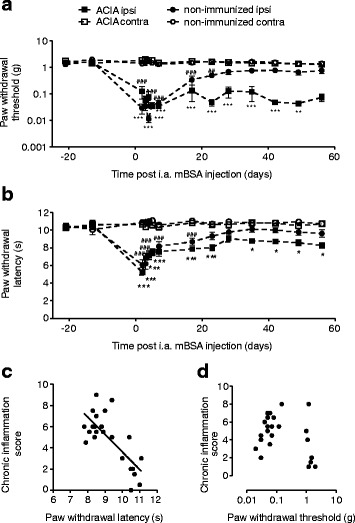
Mechanical allodynia and thermal hyperalgesia in ACIA and control-treated mice. Paw withdrawal thresholds (**a**) and latencies (**b**) were determined for both hind paws at the time points indicated using von Frey filaments (**a**) and the Hargreaves test (**b**), respectively. ACIA mice were immunized with s.c. mBSA and bovine collagen II, and arthritis was induced by i.a. mBSA in the ipsilateral joint (*open squares*), PBS was injected into the contralateral joint (*black squares*). Control animals only received s.c. adjuvant and i.a. mBSA (*open circles*) or PBS contralaterally (*black circles*). Data are represented as means ± SEM. Statistical analysis was performed using the two-way RM ANOVA and Bonferroni’s multiple comparison tests followed by a Bonferroni correction comparing ACIA ipsi- vs. contralateral thresholds (*P < 0.05, **P < 0.01, ***P < 0.001) and non-immunized ipsi- vs. non-immunized contralateral thresholds (#*P* < 0.05, ##*P* < 0.01, ###*P* < 0.001), *N* = 8. Inflammation scores of immunized, non-immunized, and CD8^+^-depleted mice were correlated with thermal paw withdrawal latencies (**c**) and mechanical paw withdrawal thresholds (**d**) using the Spearman correlation. Paw withdrawal latency was inversely correlated with inflammation scores (Spearman *r* = −0.6, *P* < 0.01, *N* = 24), no correlation was found for paw withdrawal thresholds and inflammation scores

Throughout the acute and chronic phases of joint inflammation (Fig. 2), ACIA mice showed significantly lower mechanical thresholds in the von Frey test in ipsilateral (i.a. mBSA) compared to contralateral (i.a. PBS) hind limbs (Fig. 2a). Animals that were not previously immunized, but received only i.a. ipsilateral mBSA (induction-only), showed a similar difference during the acute phase of inflammation; however, this effect decreased as the inflammation subsided (Fig. 2a).

As shown in Fig. 2b, in ACIA mice, the ipsilateral paw withdrawal latencies to heat were significantly lower than the contralateral ones at all time points. In induction-only animals, they were also significantly lower during early joint inflammation, but not at later time points (days 23–56).

The paw withdrawal latencies elicited by heat significantly decreased with increasing severity and destruction scores (Fig. 2c). In contrast, paw withdrawal thresholds elicited by mechanical stimuli did not show a correlation with parameters of inflammation or destruction (Fig. 2d).

In summary, the ACIA protocol induced hyperalgesia and allodynia for at least 60 days after arthritis induction and appears therefore suitable to study chronic pain conditions in arthritis.

### Opioid receptor antagonist exacerbates hyperalgesia

To investigate the role of endogenous opioids, we examined the effect of the opioid receptor antagonist NLXM. This compound does not cross the blood brain barrier [30] so that centrally mediated effects can be excluded.

**Fig. 3:**
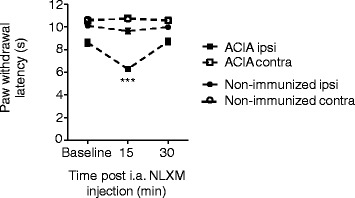
Hyperalgesia is modulated by opioid receptor antagonist. The effect of NLXM on the paw withdrawal latency was determined >day 60 after i.a. injections. Data are represented as means ± SEM. Thresholds in ACIA-mice were significantly reduced ipsilaterally at 15 min after NLXM injection as compared to baseline thresholds (Friedman and Dunn’s test, ^§§§^ *P* < 0.001), *N* = 7–8 mice. No effect was observed in the ipsilateral paw of non-immunized animals or in the contralateral paws of both groups (one-way RM ANOVA and Bonferroni’s multiple comparison test, *P* > 0.05)

ACIA and non-immunized mice received ipsilateral i.a. NLXM in the chronic stage of arthritis (>day 60). Following NLXM treatment, induction-only mice showed no differences in paw withdrawal latencies to mechanical stimulation between ipsi- and contralateral sides, whereas paw withdrawal latencies to heat were significantly reduced in ipsilateral limbs of ACIA mice as compared to baseline thresholds (Fig. 3). Thus, endogenous opioids seem to decrease heat hyperalgesia in the chronic stage of our arthritis model.

### CD8^+^ cells contribute to opioid action in chronic arthritis

**Fig. 4:**
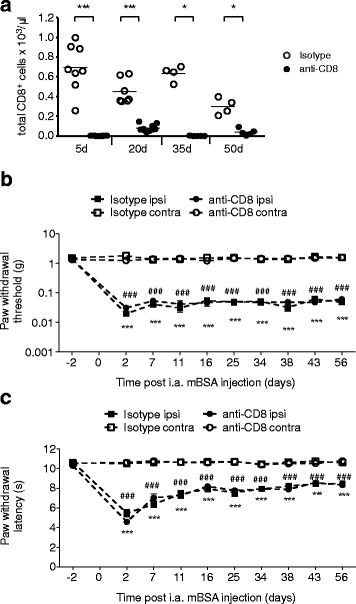
Mechanical allodynia and thermal hyperalgesia in CD8^+^-depleted arthritic mice. Prior to arthritis induction, CD8^+^ cells were depleted by repeated i.p. injections of anti-CD8 antibodies (clone 2.43) until the end of the observation period. Control mice received the isotype-matched control antibody. **a** Success of CD8^+^ depletion was confirmed in the peripheral blood by flow cytometry. Paw withdrawal thresholds and paw withdrawal latencies of CD8^+^-depleted or isotype-treated arthritic mice were determined during the onset of chronic ACIA at the time points indicated in the ipsi- and contralateral paws using von Frey filaments (**b**) and Hargreaves test (**c**), respectively. Data are represented as means ± SEM. Statistical analysis was performed using the two-way RM ANOVA and Bonferroni’s multiple comparison test followed by a Bonferroni correction comparing ipsi- vs. contralateral sides of animals receiving anti-CD8 (***P < 0.001) or a control antibody (^###^ *P* < 0.001); no significant differences were observed between ipsilateral thresholds of anti-CD8 and isotype control antibody-treated animals or between the contralateral paws of the two treatment groups, *N* = 8

The role of CD8^+^ T cells in chronic arthritis is controversial [31, 32]. We depleted these cells 3 days before i.a. injection of mBSA with a CD8-specific antibody and repeated this treatment every 9 days. Depletion efficacy was continuously surveyed by counting CD8^+^ cells in the peripheral blood by flow cytometry and was compared to mice receiving an isotype-matched control antibody. Animals receiving the anti-CD8 antibody displayed very low numbers of CD8^+^ T cells in comparison to mice treated with the control antibody at all time points (Fig. 4a). There were no significant differences in baseline nociceptive thresholds between CD8^+^-depleted and control animals (Fig. 4b, c).

**Fig. 5:**
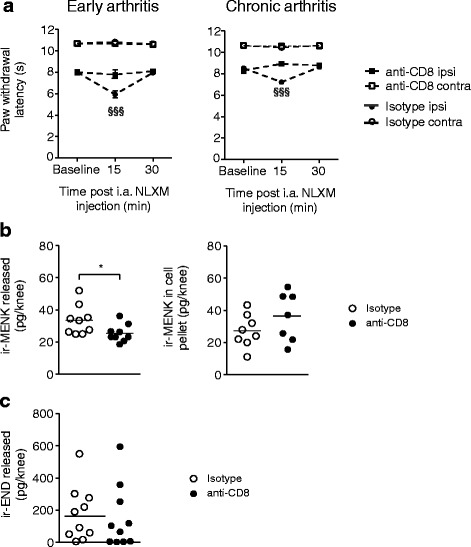
Opioid actions in normal and CD8^+^-depleted arthritic mice. **a** The opioid antagonist NLXM was injected ipsilaterally in the early (*left panel*, d16) or late chronic phase (*right panel*, >d60) of arthritis. Paw withdrawal latencies are represented as means ± SEM. Paw withdrawal latency in mice that were not depleted of CD8^+^ cells was significantly reduced ipsilaterally at 15 min after NLXM injection as compared to baseline thresholds (one-way RM ANOVA and Bonferroni’s multiple comparison test followed by Bonferroni correction,^§§§^ *P* < 0.001), *N* = 8 mice. No effect was observed in the ipsilateral paw of CD8^+^-depleted animals (early phase: one-way RM ANOVA and Bonferroni’s multiple comparison test followed by Bonferroni correction, *P* > 0.05; chronic phase: Friedman and Dunn’s test, *P* > 0.05) or in the contralateral paws of all groups (one-way RM ANOVA and Bonferroni’s multiple comparison test, *P* > 0.05). **b**, **c** The joint cells of ipsilateral arthritic knee joints was analyzed for opioid peptide release. Met-enkephalin immunoreactivities (ir-MENK, **b** *left panel*) and beta-endorphin immunoreactivities (ir-END, **c**) were assessed in the supernatant of cells, *N* = 9–10 and in the cell pellet (ir-MENK, **b** *right panel*). Statistics were performed using the Mann-Whitney *U* test to compare the effect of anti-CD8 vs. isotype matched antibody, *P > 0.05

Both groups received i.a. NLXM either 16 or 60 days after arthritis induction (Fig. 5a). At both time points, arthritic mice treated with isotype-control antibody showed significantly decreased thermal thresholds following NLXM injection, indicating exacerbated hyperalgesia. In CD8^+^-depleted animals, however, NLXM had no effect. These findings suggest an involvement of CD8^+^ cells in the fine tuning of inflammatory pain by the release of endogenous opioids at both stages. The amount of Met-enkephalin released from joint cells of CD8^+^-depleted animals was significantly lower than in isotype control-treated animals (Fig. 5b left panel), while cellular contents showed no statistically significant differences (Fig. 5b right panel). The amount of released beta-endorphin did not differ between the treatment groups (Fig. 5c).

### Impact of CD8^+^ cells on joint inflammation and destruction

**Fig. 6:**
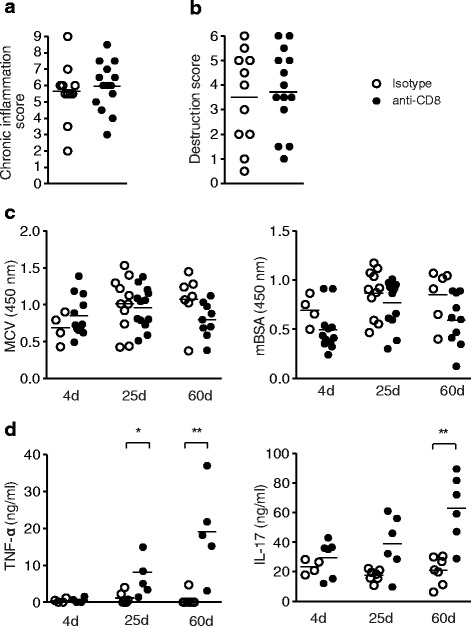
CD8^+^-depletion affects serum levels of pro-inflammatory cytokines in arthritic mice. **a**, **b** Score for inflammation and destruction in chronic phase of ACIA in CD8^+^-depleted animals or isotype-treated mice. **c** Serum levels of ACPA- and mBSA-specific IgGs in arthritic mice treated with anti-CD8 or the isotype-matched antibody (*left*: anti-MCV mutated citrullinated vimentin, *right*: mBSA). **d** TNFα and IL-17 levels are elevated in later stages of arthritis in CD8^+^-depleted arthritic mice. Statistical analyses were performed using the Mann-Whitney *U* test; *P < 0.05, **P < 0.01

In accordance with our previous results, both control antibody-treated as well as CD8^+^-depleted mice developed chronic arthritis in the later observation phases (Fig. 6a, b). Mean scores of inflammation (Fig. 6a) or destruction (Fig. 6b) did not differ statistically between the groups. Also, serum levels of arthritis markers like ACPA or antibodies against the inducing agent mBSA were not different between CD8^+^-depleted and control animals (Fig. 6c). However, serum levels of the pro-inflammatory cytokines TNFα and IL-17 were significantly increased in CD8^+^-depleted mice in the chronic stages of the disease (Fig. 6d).

## Discussion

The present study addressed an important link between the nervous and immune systems in chronic inflammatory arthritis. Given that inflammation and opioids modulate the sensitivity towards painful stimuli [18], we examined arthritis-related mechanical and thermal nociceptive responses as well as the effects of peripheral opioid receptor blockade and lymphocyte depletion. Our findings indicate that CD8^+^cells, usually considered harmful, may play a protective and analgesic role by regulating pro-inflammatory cytokines and endogenous opioid peptides in chronic stages of arthritis. In addition, we found that the knee joints of ACIA mice >60 days after onset of the disease displayed higher scores of inflammation as compared to induction-only animals that had only received i.a. mBSA. Ongoing joint inflammation in the ACIA model was accompanied by persistent mechanical allodynia and thermal hyperalgesia throughout the entire observation period (i.e., 2 months) although changes in thermal nociceptive thresholds were relatively modest. Of note, while thermal nociceptive behaviours correlated well with scores of inflammation, this was not the case for mechanical nociceptive thresholds. Apparently, more detailed measurements on mechanical nociceptive behaviors (e.g., weight bearing, paw pressure) need to be performed in future studies.

It is currently not clear if plastic changes in the nociceptive system during acute inflammation are different from those during chronic immune-mediated diseases such as RA. Our goal was to identify possible therapeutic targets for chronic pain associated with RA. Therefore, we needed to know whether ACIA could be used to assess pain. Because pain parameters have not been carefully studied in models of RA, we used two different tests to measure mechanical (von Frey hairs) and thermal hyperalgesia (Hargreaves test).

In induction-only animals, the nociceptive behaviors observed after i.a. mBSA injection returned to control levels (as determined in the PBS-injected contralateral side) after 49 days with respect to mechanical allodynia, and after 23 days for thermal hyperalgesia. Similar to previous models of antigen-induced arthritis (AIA) in mice [33], thermal hyperalgesia was strongest during the first week after induction of ACIA. However, while AIA-mice showed normal thermal thresholds after 21 days, the thresholds remained lowered in ACIA until the end of the observation period. In Lewis rats with AIA, thermal thresholds remained reduced for 21 days [34]. Nociceptive thresholds in mice with collagen-induced arthritis (CIA)-mice were studied using different tests and can therefore not be directly compared with the present findings. For example, knee pain was measured in C57BL/6 mice with CIA as weight-bearing on the hind limbs using an incapacitance meter [35] and developed later than in AIA and ACIA; it was detectable only after approximately 15 days and then became constantly stronger until the end of the observation period [35]. We found a strong correlation between thermal hyperalgesia and scores of chronic inflammation and joint destruction, indicating that the combination of AIA and CIA results in a “pain” phenotype with a fast onset and long duration, representing a novel and more appropriate tool to investigate chronic pain.

The local injection of NLXM into the mBSA-treated knee produced a short lasting but significant increase in thermal hyperalgesia in ACIA but not in non-immunized mice. This indicates the liberation of opioid peptides in chronically inflamed tissue and extends our knowledge gained from acute inflammatory models [24, 36, 37]. Importantly, no NLXM effect was detectable when the inflammation had resolved, as in non-immunized mice. This supports the findings of several previous studies demonstrating that immune cells can release opioid peptides locally under inflammatory conditions [38]. In the absence of such cells, no liberation of opioid peptides seems to take place. Because mechanical thresholds were too low to expect significant decreases after NLXM administration, we did not test this. Again, more detailed measurements on mechanical nociceptive behaviors (e.g., weight bearing, paw pressure) need to be performed in future studies.

Our next question was to investigate the role of CD8^+^ cells in chronic ACIA with respect to joint destruction and pain. Due to their cytotoxic capacity, CD8^+^ cells (CTLs) contribute to tissue destruction in a variety of physiologic and pathophysiologic processes. The damage caused by these cells can be strong, as has been reported for autoimmune diseases such as diabetes mellitus type 1, systemic lupus erythematosus, psoriasis vulgaris, and multiple sclerosis [39]. This suggested that depletion of CTLs might be beneficial in a disease model characterized by inflammation, tissue destruction, and pain. In ACIA, however, the majority of parameters investigated (autoantibody formation, chronic inflammation, and nociceptive behaviours) did not improve after CD8-antibody treatment, in accordance with previous studies [40, 41].

The histological evaluation of knee sections for signs of chronic inflammation and destruction revealed no significant differences between CD8^+^-depleted and non-depleted ACIA-mice. This is in accordance with findings obtained in proteoglycan aggrecan-induced arthritis in BALB/c mice [42]. Lower arthritis scores were observed after CD8^+^ cell-depletion in mercuric chloride-induced arthritis in rats [41]. However, mercuric chloride does not lead to chronicity of the disease and these findings cannot be directly compared to the situation in chronic ACIA.

The role of CD8^+^ cells is controversial. Indeed, they can also attenuate the clonal expansion of activated CD4^+^ T cells [43, 44], secrete the anti-inflammatory cytokine IL-10 [45, 46], or contribute to the downregulation of Th17 cells [47]. In line with this notion, we found increased levels of the pro-inflammatory cytokines TNFα and IL-17 in the absence of CD8^+^ T cells in ACIA. The blockade of IL-17 can lead to decreased joint inflammation and therefore, IL-17 is discussed as a potential therapeutic target in RA [48]. In the K/BxN mouse model, the development of spontaneous chronic arthritis does not seem to be Th17-driven, as levels of IL-17 were below detection limit in depleted and non-depleted animals [40]. In contrast, the titer of IL-17 in ACIA strongly suggests that such cells are present. This may further explain the differences observed after CD8^+^ depletion comparing the two models. In CD8^−/−^ mice, the regulatory function of CD8^+^ cells became apparent after re-challenge with the antigen followed by more severe CIA compared to CD8^+/−^ mice [49]; this resembles our findings more closely.

In agreement with other arthritis models discussed above [40–42, 49], no changes of the serologic autoantibody titers were detected after CD8^+^-depletion in ACIA.

The depletion performed in the present and in other studies eliminated cytotoxic and regulatory CD8^+^ T cells alike, a fact that could affect the experimental outcome in several ways. The most pronounced consequences of CD8^+^-depletion in several other arthritis models are related to the deletion of their regulatory functions. The K/BxN mouse model of spontaneous chronic arthritis seems to be an exception and may represent a good model to study the deleterious effects of CD8^+^ cells. It is obvious that different arthritis models differ not only in their experimental procedure (e.g., inducing agents, time points of induction and cell depletion) but also in their immune responses.

The basal nociceptive responses measured in ACIA, mechanical allodynia, and thermal hyperalgesia, remained unaffected by CD8^+^-depletion. To the best of our knowledge, there are no previous studies investigating nociception after CD8^+^-depletion in chronic arthritis. In models of acute inflammation, it was shown that endogenous opioid-mediated analgesia depends on CD4^+^ T lymphocytes [18, 22]. In our study, CD8^+^-depleted ACIA animals showed no decrease in their paw withdrawal latencies after NLXM injection. This indicates an impaired opioid system function in the absence of CD8^+^ cells. Additional studies in order to evaluate the release from other cell types are needed.

CD8^+^-depletion also leads to changes in the inflammatory milieu in ACIA, e.g., the elevation of pro-inflammatory cytokines, which could trigger the regulated release of Met-enkephalin, in agreement with calcium-dependent opioid peptide release induced by IL-1β and IL-6 [50]. Our data indicate that CD8^+^-depletion might selectively affect Met-enkephalin but not beta-endorphin liberation. These results are in line with previous studies showing that CD4^+^ T lymphocytes as well as CD8^+^ T lymphocytes produce predominantly enkephalins in mice [22, 51]. Thus, the selective reduction of enkephalin release may be explained by the depletion of this predominant source of enkephalin.

## Conclusions

In summary, using our chronic arthritis model, we could demonstrate for the first time the development of chronic pain and the correlation of joint inflammation and hyperalgesia. Additionally, our data suggest a beneficial role of CD8^+^ lymphocytes in endogenous opioid-mediated analgesia.

## Abbreviations

ACIA: Antigen- and- collagen-induced arthritis
ACPA: Anti-citrullinated peptide/protein antibodies
AIA: Antigen-induced arthritis
CFA: Complete Freund’s adjuvant
CIA: Collagen-induced arthritis
CTLs: Cytotoxic T cells
EIA: Enzyme immunoassay
IFA: Freund’s incomplete adjuvant
IL-17: Interleukin 17
mBSA: Methyl bovine serum albuvin
MCV: Mutated citrullinated vimentin
NLXM: Naloxone-methiodide
PTx: *Bordetella pertussis* toxin
RA: Rheumatoid arthritis
RIA: Radioimmunoassay
TNFα: Tumor necrosis factor alpha
